# Sediment Core DNA‐Metabarcoding and Chitinous Remain Identification: Integrating Complementary Methods to Characterise Chironomidae Biodiversity in Lake Sediment Archives

**DOI:** 10.1111/1755-0998.14035

**Published:** 2024-10-21

**Authors:** Lucas André Blattner, Pierre Lapellegerie, Colin Courtney‐Mustaphi, Oliver Heiri

**Affiliations:** ^1^ Department of Environmental Sciences, Geoecology University of Basel Basel Switzerland; ^2^ Natural History Museum Basel Basel Switzerland

**Keywords:** Chironomidae, lake sediments, morphogroups, Paleolimnology, SedDNA

## Abstract

Chironomidae, so‐called non‐biting midges, are considered key bioindicators of aquatic ecosystem variability. Data derived from morphologically identifying their chitinous remains in sediments document chironomid larvae assemblages, which are studied to reconstruct ecosystem changes over time. Recent developments in sedimentary DNA (sedDNA) research have demonstrated that molecular techniques are suitable for determining past and present occurrences of organisms. Nevertheless, sedDNA records documenting alterations in chironomid assemblages remain largely unexplored. To close this gap, we examined the applicability of sedDNA metabarcoding to identify Chironomidae assemblages in lake sediments by sampling and processing three 21–35 cm long sediment cores from Lake Sempach in Switzerland. With a focus on developing analytical approaches, we compared an invertebrate‐universal (FWH) and a newly designed Chironomidae‐specific metabarcoding primer set (CH) to assess their performance in detecting Chironomidae DNA. We isolated and identified chitinous larval remains and compared the morphotype assemblages with the data derived from sedDNA metabarcoding. Results showed a good overall agreement of the morphotype assemblage‐specific clustering among the chitinous remains and the metabarcoding datasets. Both methods indicated higher chironomid assemblage similarity between the two littoral cores in contrast to the deep lake core. Moreover, we observed a pronounced primer bias effect resulting in more Chironomidae detections with the CH primer combination compared to the FWH combination. Overall, we conclude that sedDNA metabarcoding can supplement traditional remain identifications and potentially provide independent reconstructions of past chironomid assemblage changes. Furthermore, it has the potential of more efficient workflows, better sample standardisation and species‐level resolution datasets.

## 1 Introduction

Freshwater lakes are vital ecosystems that support an array of life forms and provide essential resources for human societies (Garcés‐Pastor et al. 2022; Kerdy, Chiquet, and Schibler 2019). These ecosystems are not only sensitive to contemporary environmental changes but also bear the records of past ecosystem trajectories, preserved in their sediments by the remains of organisms that lived in the past (e.g., Li et al. 2023; Stivrins et al. 2016). To gain insight into the complex interplay between past and present ecosystem dynamics over decades to millennia, Paleolimnology, a discipline that investigates ancient environmental conditions by studying lake sediments, provides a valuable window into the past (Battarbee and Bennion 2011; Covert and Medeiros 2021). At the same time, well‐preserved remains of organisms in lake surface sediments, such as recently deposited exoskeletons of invertebrates or valves of diatoms, can be analysed to assess the modern distribution of biota in lakes. For example, much of what is known about the distribution of the larvae of Chironomidae, also known as non‐biting midges, or diatoms in small European mountain lakes is based on the analyses of their remains in recently deposited lake surface sediment samples (e.g., Heiri and Lotter 2010; Lotter et al. 1998).

Paleolimnological approaches rely on identifying indicators of past environmental conditions in the lake sediment record, i.e., abiotic and biotic tracers and microfossils (e.g., Smol 2009), as well as more recently ancient DNA (e.g., Domaizon et al. 2017; Ficetola et al. 2018). Sediment cores encapsulate the history of a freshwater lake and its inhabitants and serve as repositories of information documenting environmental conditions over time (Ellegaard et al. 2020; Jiménez et al. 2019). Among the diverse biota found and studied in sediment cores, the dipteran family Chironomidae plays a pivotal role and serves as a model system for environmental reconstruction (Eggermont and Heiri 2012; Engels et al. 2020; Luoto, Kotrys, and Płóciennik 2019; Medeiros et al. 2022). In both their larval and adult stages, these insects are integral components of aquatic food webs and are sensitive indicators of environmental change (Cortelezzi et al. 2020; Lencioni, Marziali, and Rossaro 2012; Marziali, Lencioni, and Rossaro 2006). Their sensitivity to environmental conditions makes them ideal subjects for studying ecological responses to changing temperatures (Heiri et al. 2011; Kotrys et al. 2020; Luoto, Kotrys, and Płóciennik 2019), nutrient concentrations (e.g., Brooks, Bennion, and Birks 2001) or oxygen availability (e.g., Quinlan and Smol 2001a; Verbruggen et al. 2011). Furthermore, chironomids serve as relevant environmental indicators for approaches to assess contemporary ecological conditions in lakes (Jyväsjärvi, Aroviita, and Hämäläinen 2014; Rossaro et al. 2006).

The identification and classification of Chironomidae based on sediment core analysis require the isolation of their remains from sediments followed by microscopic examination that relies on distinguishing morphological characteristics of the chitinised head capsules preserved in the sediment record (Brooks, Langdon, and Heiri 2007), often over hundreds to thousands of years (e.g., Bennike et al. 2023; Bolland et al. 2022). In addition to chironomids, the chitinous remains of other aquatic invertebrates that are common in the samples are often identified as well, although generally to a less detailed taxonomic resolution (e.g., genus to order level; Courtney‐Mustaphi et al. 2024). However, despite its constant advancements, this traditional approach still has limitations, such as challenging identification due to selective preservation of morphological features, generally difficult Chironomidae larvae identification, and the complexity of distinguishing closely related taxa (Brooks 2006; Czechowski et al. 2020; Heiri and Lotter 2010). This results in the identification of chironomids to species‐ or genus‐level morphotypes (Brooks 2006; Heiri et al. 2011) and can lead to varying taxonomic resolutions and challenges in reaching bioindication‐derived conclusions (Bailey, Norris, and Reynoldson 2001; Jones 2008).

Advances in molecular biology, specifically the utilisation of metabarcoding techniques in combination with the analysis of sediment DNA (sedDNA), have shown to offer the potential to enhance the ability to explore the diversity of species and supplement traditional approaches (Barouillet et al. 2022; Capo et al. 2021; Li et al. 2023). SedDNA allows for the direct extraction and sequencing of genetic material from sediment samples, overcoming the need to isolate chitinous remains followed by challenging morphological identification, hence enabling the detection of taxa that might otherwise remain undetected. Despite recent developments, i.e., the application of sedDNA approaches to different taxonomic groups such as foraminifera (Pawlowska, Pawlowski, and Zajaczkowski 2020), plants (Garcés‐Pastor et al. 2022), fish and mussels (Thomson‐Laing et al. 2023) or crustaceans (copepods; Nakane et al. 2023), the suitability of sedDNA metabarcoding to detect Chironomidae species remains, to our current knowledge, largely unexplored.

Therefore, this study aims to investigate the applicability and potential of sediment core DNA metabarcoding techniques to supplement data derived from morphological identifications of chitinous Chironomidae remains. Furthermore, we seek to address the primer bias effect, known as one of the major challenges in metabarcoding studies (Brantschen et al. 2022; Elbrecht and Leese 2015; Vamos, Elbrecht, and Leese 2017), by examining invertebrate‐universal and chironomid‐specific PCR primer sets. Overall, we develop a first assessment of the comparability among morphological remains identification and sedDNA analysis, and we evaluate their performance in identifying chironomid assemblage changes between the surface sediment cores obtained from a Swiss lowland lake. We combine and systematically compare our sedDNA results with the morphological chironomid remains data derived from the conventional lake sediment analysis. Furthermore, we seek to explore the potential of an integrated approach based on combining the two methods to study both the present and historical dynamics of Chironomidae populations over time in future research. Such integrated studies would potentially provide more refined insights into chironomid responses to environmental drivers and stressors, both at present and in the past, and allow for more detailed assessments of their potential future response to environmental change.

## 2 Materials and Methods

### 2.1 Study Site and Sediment Coring

**FIGURE 1:**
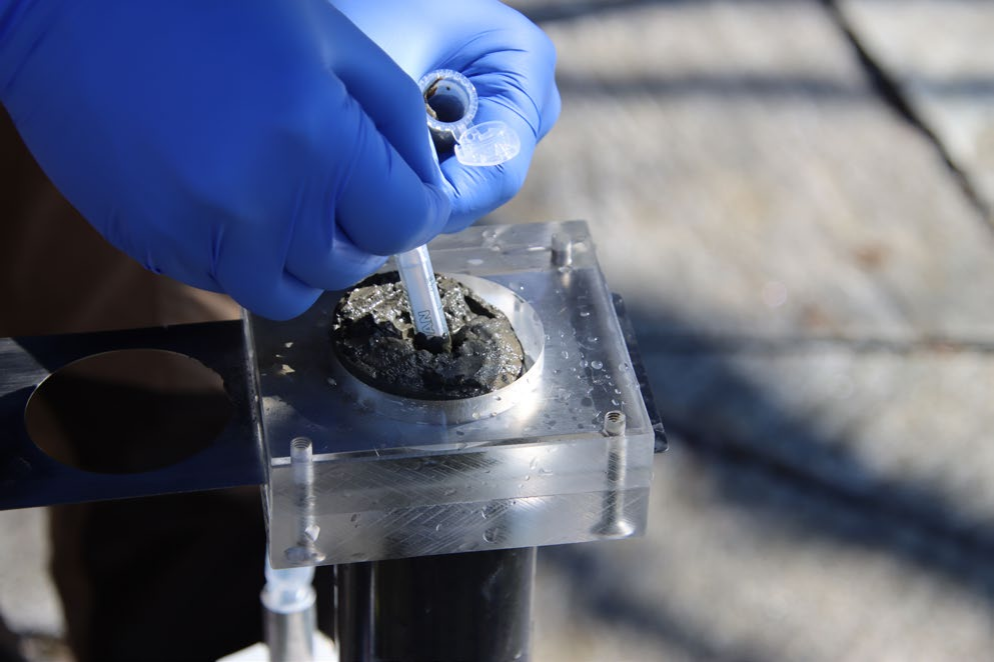
SedDNA sampling from a vertical extruder with a sterile syringe and a core cutting tool. The sediment core layer samples were taken from the centre of each core section to avoid the potentially contaminated sediments along the tube wall.

Lake Sempach is a eutrophic hardwater lake on the Swiss Plateau (Canton of Lucerne) with a surface area of 14.4 km^2^, a mean depth of 44 m and a maximum depth of 87 m (Gächter and Wehrli 1998). To limit the extent of anoxic conditions, the hypolimnion has been artificially oxygenated since 1984, and the lake has been monitored intensively since (e.g., Gächter and Wehrli 1998; Müller and Stadelmann 2004; Su et al. 2022). Three surface sediment cores (SS1, SS2 and SS3; Table 1) were sampled in February 2022 at different water depths from a boat using a gravity‐based coring system USC06000 (UWITEC, Austria) and PVC tubes with a 60 mm internal diameter. Immediately after sampling, the sediment cores were brought to the shore and extruded in a vertical position using a core cutter (Glew, Smol, and Last 2001). For sedDNA metabarcoding, three 15 mm thick layers per core were sampled at the water–sediment interface, approximately in the middle and at the bottom of the 21–35 cm long cores (Table 1). Sediments were extruded by pushing the core tube unto a piston inserted at the bottom of the core and were collected by a sediment subsampler fixed unto the top of the coring tube. The subsampler allowed sediment slices to be separated from the rest of the core by horizontally cutting sediment slices with a thin metal plate. For sedDNA analysis, 1 mL of extruded sediment per depth layer was sampled at the centre of each core section to avoid potential contamination from the tube wall area (Figure 1). We used tip‐cut, sterile, single‐use 5 mL syringes (B.Braun Medicals, Switzerland) to sample the sediment and transferred it directly to PCR‐grade 5 mL Safe‐Lock tubes (Eppendorf, Germany) (Figure 1). Before and after each subsampling, instruments and cutter were cleaned with bleach (sodium hypochlorite, 13%) and rinsed with ethanol (80%) to prevent sedDNA cross‐contamination between the samples. To monitor potential sample contamination and include a field‐negative control for each subsample, we positioned an empty, open 5 mL tube approximately 5 cm next to the exposed sediment core for the duration of subsampling to potentially collect the ambient contaminating environmental DNA (eDNA). All sedDNA samples were stored on dry ice immediately and transferred to −20°C upon arrival at the laboratory until further processing. The remaining sediment of approximately 47 cm^3^ sediment per slice for each sampling depth was collected and stored in plastic bags for the isolation and identification of chitinous chironomid larvae remains.

**TABLE 1:** Lake Sempach sediment core sampling locations.

ID	Water depth (m)	Lat (WGS84)	Lon (WGS84)	Core length (cm)	DNA top	DNA middle	DNA bottom
SS1	16.0	47.143850	8.177600	21	0–2	8–10	18–20
SS2	22.1	47.143600	8.177000	26	0–2	10–12	22–24
SS3	62.0	47.141300	8.167617	35	0–2	19–20	27–28

### 2.2 Isolation and Identification of Chitinous Remains

We analysed 6–45 g of wet sediment per sample, depending on the amount needed to reach a minimum count of 50 chironomid head capsules, which is a common procedure in palaeoecological analysis of chironomid remains (Heiri and Lotter 2001; Larocque 2001; Quinlan and Smol 2001b). Moreover, also chitinous remains of other aquatic invertebrates were isolated and analysed. The preparation of the samples followed conventional methods outlined in Brooks, Langdon, and Heiri (2007). Samples were treated with potassium hydroxide (KOH; 30%) at 70°C for 30 min before being washed through a 100 μm mesh‐size sieve with water. Subsequently, the chironomid head capsules and other invertebrate remains were hand‐picked with forceps from a Bogorov tray using a stereomicroscope at 20–50× magnification. Thereafter, all head capsules were prepared on microscope slides and embedded in Euparal mounting medium. We identified the chironomid and other aquatic invertebrate remains at the highest taxonomic resolution possible with a compound microscope (100–400× magnification) following Andersen, Cranston, and Epler (2013), Brooks, Langdon, and Heiri (2007) and Schulze (1993). Other aquatic invertebrate remains were identified to a coarse (order, family and genus) taxonomic resolution and included Chaoboridae, Ceratopogonidae, Ephemeroptera, Trichoptera, Plecoptera and Simuliidae (identified based on Courtney‐Mustaphi et al. 2024). Remains of Cladocera were identified following Szeroczyfiska and Sarmaja‐Korjonen (2007), Bryozoans based on Wood and Okamura (2005) and oribatid mites with Solhøy (2001). Raw chitinous remains counts are documented in Table S2.

### 2.3 Sediment DNA (sedDNA) Extraction and Purification

To choose the best method combination for sedDNA extraction, we first compared possible technical differences in DNA yield due to the amount of sediment and the extraction method. In the laboratory, we homogenised each sediment sample by vortexing after thawing. We took three 250 mg and three 500 mg extracts of the 1 mL wet samples of each core and layer using cut‐tip 1000 μL pipette tips and a micro scale (Merck, Germany), which resulted in a total of 54 subsamples (three cores, three layers per core and three 250 and 500 mg subsamples of each core layer). Subsequently, we extracted total DNA out of these subsamples with (A; PowerSoil) the DNeasy PowerSoil Pro kit (Qiagen, Germany), (B; PowerSoilPhos) the same kit but with an addition of 1 M phosphate buffer as recommended by Direito, Marees, and Röling (2012) and (C; FastDNA) with the FastDNA Spin Kit for Soil (MP Biomedicals, California USA). The extraction kits were used according to the manufacturer protocols, and an additional Proteinase K digestion was performed during the lysis steps. In the case of the two PowerSoil Pro kit treatments, we added 20 μL Proteinase K [20 mg/mL] (Qiagen, Germany) to the 800 μL of buffer CD1 (protocol step 1). We incubated the solution containing the sediment in PowerBead Pro Tubes at 56°C for 2 h before continuing strictly according to the manufacturer's manual. The FastDNA Spin kit was modified at the first step of the protocol by adding the same amount and concentration of Proteinase K to the sediment solution and incubating the samples with the same conditions before continuing according to the manual and homogenisation on the FastPrep instrument. All DNA eluates, including the field negative controls, were then quantified using a Qubit fluorometer and the high‐sensitivity dsDNA kit (ThermoFisher, USA; Table S5).

The comparison between the kits revealed minor eluate DNA concentration differences between the two PowerSoil Pro kit approaches but negligible increased DNA yield with the FastDNA kit (Figure S1A). Therefore, we proceeded with the PowerSoil Pro and FastDNA extracted samples (see Table S5). The amount of sediment showed a more pronounced effect on the results with an overall higher median DNA eluate concentration in the 500 mg samples (Figure S1B), which were then further processed to enable an exploratory comparison at the exact sequence variant (ESV) level (see Section 3.2).

### 2.4 SedDNA CO1 Amplicon Data Generation

Differences in taxonomic coverage due to biases from the choice of primer combinations are a major challenge in metabarcoding studies (Bohmann et al. 2022; Elbrecht and Leese 2015; Zizka, Geiger, and Leese 2020). Therefore, we compared the commonly used freshwater invertebrate universal primer combination fwhF2/fwhR2n targeting a 254 bp long CO1 fragment (hereon abbreviated FWH) (Leese et al. 2021; Elbrecht et al. 2019; Vamos, Elbrecht, and Leese 2017), with a newly developed more Chironomidae‐specific primer combination CH181F: 5′‐TAA TYT TYT TYA TRG TNA TRC C‐3′ and CH181R: 5′‐CCN GTI CCI GCH CCR TTT TC‐3′ (hereon abbreviated CH). The new primer set was designed by aligning Cytochrome c oxidase I (MT‐CO1) gene fragments obtained from 89 Chironomidae sequences downloaded from NCBI GenBank and BOLD (accessions are available in Figure S3 and Table S4). The alignments were computed by the MAFFT (Katoh and Standley 2013) algorithm implemented in Geneious Prime 2023.2.1 (https://www.geneious.com). The new PCR primer combination was then designed by hand, targeting a relatively short 181 bp fragment to improve suitability for the amplification of degraded DNA (Liu et al. 2020; Wei, Nakajima, and Tobino 2018), and located at the 5′ end of the gene to ensure good reference sequence database coverage for species identification. To ensure that the obtained CO1 fragment has enough taxonomic resolution, we calculated the percental distances between the 139 bp long Chironomidae species insert sequences previously aligned in the primer design process with the *dist.dna* function implemented in the ape R package v5.7.1 (Paradis and Schliep 2019). The results showed interspecific distances between all species of at least 8% (Figure S3 and Table S4). This indicates valid species‐level sequence assignment of Chironomidae species below that threshold using our newly designed primer combination. Microsynth AG (Balgach, Switzerland) finally synthesised both primer sets with standard Illumina amplicon library overhang adapter sequences.

The sedDNA metabarcoding PCR reactions were run in triplicates to reduce reaction‐specific PCR bias using the inhibitor tolerant UCP Multiplex PCR Kit (Qiagen). All reactions, including the three field‐negative controls, were prepared in a 25 μL total volume containing 5 μL of template DNA, 1× (6.25 μL) UCP Multiplex PCR Master Mix [4×], 0.8 μM (2 μL) forward and 0.8 μM (2 μL) reverse primer [10 μM each] and 9.75 μL of molecular grade water. Reactions were run with an initial polymerase activation at 95°C for 2 min, followed by 40 cycles of 10 s denaturation at 95°C, 30 s annealing at 48°C and an extension at 68°C for 1 min, followed by a final extension step at 72°C for 5 min. No template controls (NTC) containing molecular grade water instead of the template DNA and positive controls containing gDNA of a freshly extracted Chironomidae individual were included in each run. All PCR reactions were assessed using an agarose gel electrophoresis, and none of the control reactions showed an indication of amplification. Subsequently, the sedDNA PCR triplicates were pooled and were cleaned with AMPure XP Beads (Beckman Coulter Life Sciences, USA) using a 1.6× bead ratio according to the manufacturer protocol and eluted in 100 μL TE low EDTA buffer. The final libraries, including the field‐negatives, were prepared and indexed with a standard Nextera XT (Illumina, USA) 2‐step PCR process by the Genomics Technologies Facility (GTF) in Lausanne (Switzerland) and paired‐end sequenced on an Illumina MiSeq system with a v2 reagent kit and 2 × 150 cycles. Raw sequencing data is available on the NCBI SRA database under the BioProject Accession: PRJNA1062313 (for sample assignment, see Table S5).

### 2.5 Data Processing and Analysis

A total of 12,659,249 raw sequence reads were quality checked using FastQC v0.11.8 (Andrews 2010) and MultiQC v1.11 (Ewels et al. 2016). Forward and reverse reads were merged using *fastq_mergepairs* from vsearch v2.21.1 (Rognes et al. 2016), requiring a minimum overlap of 50 bases and allowing a maximum of 10 nucleotide differences. Primer sequences were removed with the linked primer and anchored 5′ primer options using cutadapt v3.4 (Martin 2011). Merged and primer‐trimmed reads were then quality filtered with the vsearch *fastx_filter* tool, allowing for ±10 bp sequence length deviation from the expected 136 bp (CH) or 205 bp (FWH) long CO1 amplicons and discarding reads with ≥ 1 expected error and below Q30. Quality filtered reads were then concatenated and dereplicated with vsearch *fastx_uniques* and denoised using swarm v3.1.0 (Mahé et al. 2021) with the local number of differences parameter d set to 1 and in fastidious mode to obtain so‐called zero‐radius operational taxonomic units (ZOTUs) (see, e.g., Mächler et al. 2021) or denoised exact sequence variants (ESVs). We then detected and filtered possible chimeric ESVs with the uchime3 algorithm (Edgar et al. 2011) implemented in vsearch and filtered likely pseudogenes or nuclear copies of mitochondrial DNA (so‐called numts) (Hazkani‐Covo, Zeller, and Martin 2010) by detecting stop‐codon containing sequences and removing them. To get read abundances per ESV and sample, we stringently mapped the raw reads to the final ESVs with *usearch_global* implemented in vsearch, allowing one difference between the read and the ESV (‐‐maxdiffs 1).

For taxonomic annotation of the ESVs, we first developed a curated CO1 reference sequence database. Therefore, we downloaded all available CO1 sequences belonging to Chironomidae and taxa commonly found and identified in freshwater lake habitats (82 taxonomic groups; for details, see Table S6) from NCBI GenBank and BOLD in June 2023, available on the Zenodo data repository under the DOI: 10.5281/zenodo.11527768. This reference set has a total of 1.9 Mio. sequences containing 55,410 species and 5686 higher level taxa, with an average number of sequences per species ranging from 1.3 to 78 (for details, see Table S6). The sequences were then filtered with seqkit v2.5.1 (Shen et al. 2016) to have a minimum length of 200 bp, no internal Ns and dereplicated with vsearch *fastx_uniques*. The taxonomic annotation was obtained by using the function *bold_specimens* of the bold v1.3.0 R package (Dubois and Chamberlain 2023), which serves as interface to the BOLD API in the case of BOLD, and with the function *accessionToTaxa* implemented in the taxonomizr v0.10.2 R package (Sherrill‐Mix 2023) in the case of NCBI GenBank derived sequences. Taxon names containing suspicious wording or indications of uncertainties (sp., aff., gr. or var.) were removed, and the taxonomic annotation of the respective reference sequence was reduced to the last trustable taxonomic level, e.g., species level annotation *Cladotanytarsus* gr. *mancus* was deleted, and the annotation was restricted to the genus *Cladotanytarsus*. Finally, all reference sequences were limited to the primer‐specific amplicons with an *in silico* PCR conducted using cutadapt. We trimmed the respective primer sequences, allowing for one mismatch and restricting the amplicon length to ± 10 bp. All untrimmed sequences were discarded.

The obtained final ESVs were then taxonomically annotated using the *usearch_global* algorithm implemented in vsearch, which performs a global alignment strategy. A min. identity of 90% among subject and query sequences was requested (see, e.g., Leese et al. 2021), and we accepted a max. of 5000 database hits per query sequence. The BLAST‐like output was then strictly filtered. We determined the best hits as matches having the lowest e‐value, the highest identity, the highest bit score, the lowest amount of alignment gaps and the lowest amount of mismatch positions between the query and subject sequence. If more than one best‐hit sequence with differing taxonomic annotations were present, we decided to use the lowest common ancestor (LCA) between these reference sequences as the final taxonomic annotation of the ESVs. Furthermore, we requested at least 95% sequence identity for species‐level assignments, a threshold suitable for Chironomidae and freshwater invertebrate species in general (Ekrem et al. 2018; Lin, Stur, and Ekrem 2015). All initial species assignments with < 95% matches were set back to the genus level. Raw taxonomic assignments of ESVs are documented in Tables S7 (CH) and S8 (FWH). Due to the expected low read abundance of rare species in sedDNA studies (see, e.g., discussions in Capo et al. 2021; Capo, Barouillet, and Smol 2023) and our stringent read filtering process, we allowed for low read abundance taxa detection in our study system.

### 2.6 Comparison Between Chitinous Remain Morphotypes and sedDNA Data

To compare the sedDNA‐identified taxa with the fossil morphotype identifications, we assigned the sedDNA‐derived taxonomic annotation to the equivalent fossil morphotypes based on available species‐or genus‐level information and morphotype descriptions of Chironomidae larvae (Andersen, Cranston, and Epler 2013; Brooks, Langdon, and Heiri 2007; Table S9) and other aquatic invertebrate groups. Chironomidae taxa without a morphotype assignment, i.e., without a described larva, were not included in the comparison analysis. Due to the very different nature of the abundance data derived from the two methodological approaches, i.e., fossil remain counts vs. read abundance, we transformed all abundance data to presence–absence before ordination with detrended correspondence analysis (DCA). DCAs were calculated for comparing taxa assemblage results for the chitinous remains analyses among the different cores, for comparing the species composition of the two primer combinations FWH and CH with the chitinous remains data, and for comparing the results of primer combination FWH and CH for the three cores. All data analysis steps were conducted using the R package vegan v2.6.4 (Oksanen et al. 2020) with the *decorana* function and downweighing rare taxa (iweigh = 1) for the DCAs and the tidyverse v2.0.0 package (Wickham et al. 2019) for data parsing and visualisation.

## 3 Results

### 3.1 Chitinous Remains Identifications

**FIGURE 2:**
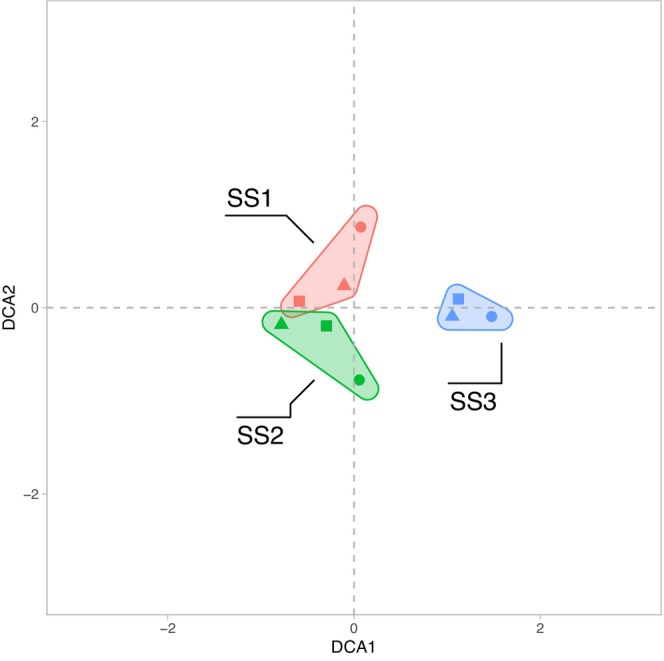
DCA of all fossil remain morphological identifications. Plot of the first two axes of a DCA (detrended correspondence analysis) ordination based on binary presence–absence data derived from chitinous Chironomidae, and invertebrate remains counts. Coloured convex hulls indicate the different cores, and the symbols represent the core layer samples (circle = bottom, triangle = middle and square = top).

We assigned the chitinous invertebrate remains to 83 different morphotypes, including 70 Chironomidae morphotypes, which were the most abundant invertebrate remains (Figure S10 and Table S11). The morphotypes Tanytarsini undifferentiated, the *Microspectra radialis*‐type and the *Micropsectra insignilobus*‐type showed the highest abundances and appeared in all three sediment cores (Figure S10). However, the relative abundances of the two *Micropsectra* morphotypes were higher in the samples of the deepest core (SS3). *Sergentia coracina*‐type is also more abundant in the deepest core in contrast to *Procladius*, *Polypedilum*, *Cladotanytarsus mancus*‐type and the *Chironomus anthracinus*‐type, which are more abundant in the shallower cores (SS1 and SS2). Results showed that approximately 31% (26) of the morphotypes appeared in all three sediment cores, 30% (25) were shared between SS1 and SS2, and the rest was either core specific or shared between SS1 or SS2 and SS3 (Figure S10 and Table S11). The relatively high proportion of shared morphotypes between the shallow‐water cores SS1 and SS2 was also evident in the corresponding DCA analysis, indicating that samples from these two cores are more similar in contrast to the deep lake core SS3 (Figure 2). However, despite the substantial similarity between SS1 and SS2, the deep‐lake core SS3 showed only three private morphotypes, whereas the other taxa were also present in the shallow water cores. The taxa unique to SS3 were *Demicryptochironomus*, *Labrundinia* and the *Rheocricotopus fuscipes*‐type, all with only a single occurrence in the samples (Figure S10 and Table S11). Overall, the number of Chironomidae remains was higher in the bottom sample of each core, with the concentration of the samples from the SS1 core being much higher than the other two cores. Apart from chironomid remains, *Daphnia* ephippia and Rhabdocoela remains were also relatively abundant in the cores but showed high variability between samples.

### 3.2 SedDNA Sequence Diversity Analysis

**FIGURE 3:**
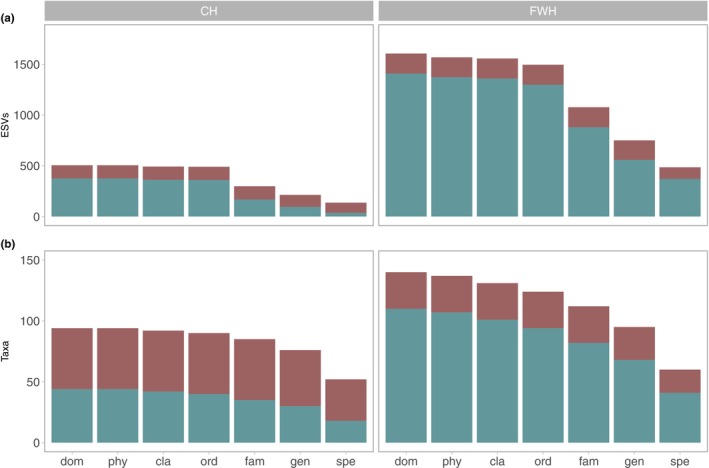
Number of ESVs and different taxa per taxonomic level and primer combination. Comparison between total ESV (A) and taxon (B) diversity among the chironomid‐specific (CH) and the invertebrate universal (FWH) primer sets. The proportion of Chironomidae‐assigned ESVs or taxa is shown in dark red, and non‐Chironomidae assigned ESVs or taxa are coloured in turquoise.

The FWH primer combination yielded a total of 3,272,928 reads, assigned to 45,343 high‐quality ESVs, out of which 1605 (3.5%; Figure 3A) were identified and taxonomically annotated to six phyla: Arthropoda (1551 ESVs), Cnidaria (6 ESVs), Chordata (5 ESVs), Mollusca (4 ESVs), Nematoda (2 ESVs) and Porifera (1 ESV) (Table S12). In contrast, the Chironomidae‐specific primer set (CH) resulted in 2,508,633 reads assigned to 11,679 ESVs with 505 (4.3%; Figure 3A) identified to three phyla, mainly Arthropoda (464 ESVs), Annelida (39 ESVs) and Chordata (2 ESVs) (Table S12). In the CH dataset, all annotated ESVs were successfully assigned at least to order level, whereas, with the universal FWH primer set, 110 ESVs remained at the class level. The FWH dataset covered a broader taxonomic spectrum composed of 22 orders, 54 families, 63 genera and 60 different species, with 30.2% of the identified ESVs (*n* = 485) assigned to species level and 46.7% (750 ESVs) to genus level. The taxonomically narrower CH primer combination resulted in 15 orders, 23 families, 21 genera and 52 different species, with 26.7% of the ESVs assigned to the species level and 42.2% to the genus level.

In contrast to the more Arthropoda universal FWH primer set, resulting in 12.3% Chironomidae ESVs (*n* = 197), and the detection of 14 Chironomidae genera and 19 Chironomidae species, the CH primer combination led to 25.7% of the identified ESVs (*n* = 130) assigned to Chironomidae with 22 genera and 33 species. Overall, the Chironomidae‐specific primer combination resulted in fewer ESVs with assignment but in a higher proportion of Chironomidae species assigned ESVs (Figure 3A) and a higher proportion of Chironomidae taxa throughout all taxonomic levels (Figure 3B).

The bottom and top layers of the shallow‐depth sediment core SS1 samples showed the highest proportion of Chironomidae assigned ESVs compared to the other cores (Figure S13A). Furthermore, the PowerSoil extraction method and the 500 mg samples resulted in a higher median amount of ESVs per sample in SS1, whereas this pattern was not consistent in cores SS2 and SS3 (Figure S13B,C).

Sequencing of the core‐specific field controls resulted in a few reads per sample. After merging and quality filtering, the field control samples of the CH‐Primer set resulted in 44 reads for SS1, 26 for SS2 and 23 for SS3. The FWH‐Primer set controls contained 69 reads for SS1, 15 for SS2 and 139 for SS3. Processing these reads resulted in two CH‐specific ESVs and 16 FWH primer‐derived ESVs. Both CH‐derived ESVs did not match any of the reference sequences. A BLAST search resulted in a possible but low‐identity match of one ESV with *Lumbricus rubellus* (a common earthworm species) present in SS1 and the other ESV with mitochondrial sequences associated with *Bos taurus* (domestic cattle) in SS2 (Table S14). Only one of the FWH field‐control ESVs showed a reference database match. The detected *Hydra virdissima* (widely distributed Hydrozoa species) appeared in all three field control samples (Table S14). BLAST searches of the other FWH‐derived field control ESVs revealed potential bacterial sequences, *Hyaloperonospora nasturtii‐aquatici* (a fungus known as an aquatic plant parasite) in sample SS2, an unspecified environmental sample and other low‐identity matches (Table S14).

### 3.3 Taxonomic Composition

**FIGURE 4:**
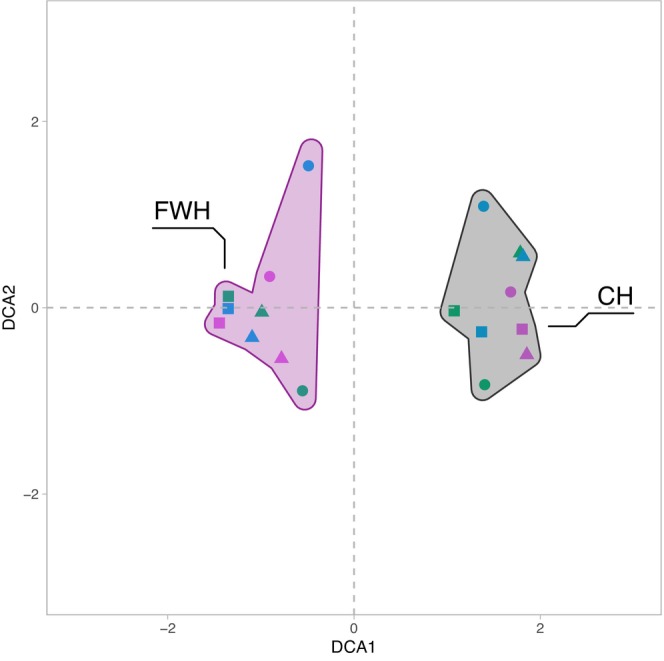
DCA ordination showing the taxonomic clustering of the sedDNA data. The ordination is based on binary presence/absence transformed read abundance data derived from all sedDNA metabarcoding taxa identifications. Coloured convex hulls indicate the invertebrate universal (FWH) and the chironomid‐specific (CH) primer sets. The symbols represent the core layer samples (circle = bottom, triangle = middle and square = top).

The two different primer sets, CH and FWH, resulted in primer‐specific overall taxa composition if all detected taxa were considered, as shown by the DCA analysis (Figure 4). Among the taxa with the highest read abundances, e.g., the Annelida genus *Tubifex* and the Chironomidae species *Cladotanytarsus mancus* (Walker, 1856), were specific to the CH primer set. In contrast, the sequence read‐rich copepod species *Mesocyclops leuckarti* (Claus, 1857) and the ostracod order Podocopida were detected with FWH only (Table S12 and Figure S15). In total, 51 Chironomidae taxa were recorded with the two primer sets. However, 26 taxa were specific to CH and 9 specific to FWH compared to 16 taxa that were detected with both primer sets simultaneously as, e.g., *Stempellinella edwardsi* Spies & Sæther, 2004 and *Cladotanytarsus atridorsum* Kieffer, 1924, both showing high read abundances (Table S12 and Figure S15). In contrast to 37 Chironomidae taxa (72.5% of the 51 total chironomid taxa) specific to a particular sediment core, only eight species appeared in all three cores (SS1, SS2 and SS3), with *S. edwardsi*‐type and *C. mancus*‐type showing the highest read abundances (Table S12 and Figure S15).

### 3.4 Chitinous Remain Versus sedDNA Morphotype Assemblages

To compare identifications based on chitinous remains with taxa assignations based on sedDNA metabarcoding, we assigned sedDNA‐derived taxa to the equivalent chitinous remains‐based morphotype (Table S9). Overall, we recorded 107 different morphotypes with three different methodological approaches, of which 78 (73%) belong to the Chironomidae family (Table S16). Among the chironomid morphotypes, we recorded 44 different species‐level morphotypes, indicating the presence of at least 44 different Chironomidae species in the dataset. Most chironomid morphotype detections (48; 63%) were recorded with the conventional approach based on morphologically identifying the chitinous remains (CHIT). These morphotypes originated from at least 29 different chironomid species.

**FIGURE 5:**
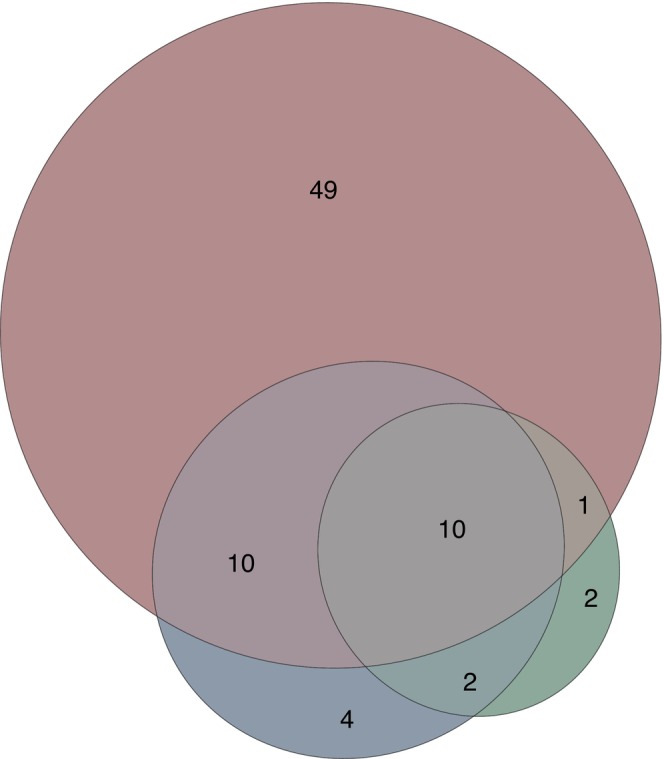
Chironomidae morphotype assemblage comparison. The Venn diagram shows the number of shared and private morphotypes among the sedDNA‐based approach (CH primer combination in blue and FWH in green) and the Chironomidae identifications based on chitinous remains (CHIT; indicated in red).

A total of 52 morphotypes were CHIT‐specific, out of which only two morphotypes (the Daphniidae genus *Ceriodaphnia* and the Platyhelminthes order Rhabdocoela) were non‐chironomid classifications not found with CH or FWH (Table S16). Seven detected morphotypes were CH‐specific, with four Chironomidae morphotypes belonging to the *Chironomus plumosus*‐type, *Macropelopia*, *Metriocnemus* and *Diamesa* (Figure 5 and Table S16). The universal primer set FWH detected 10 unique morphotypes with the two Chironomidae groups *Parametriocnemus*/*Paraphaenocladius* group and the *Polypedilum convictum*‐type (Figure 5 and Table S16).

**FIGURE 6:**
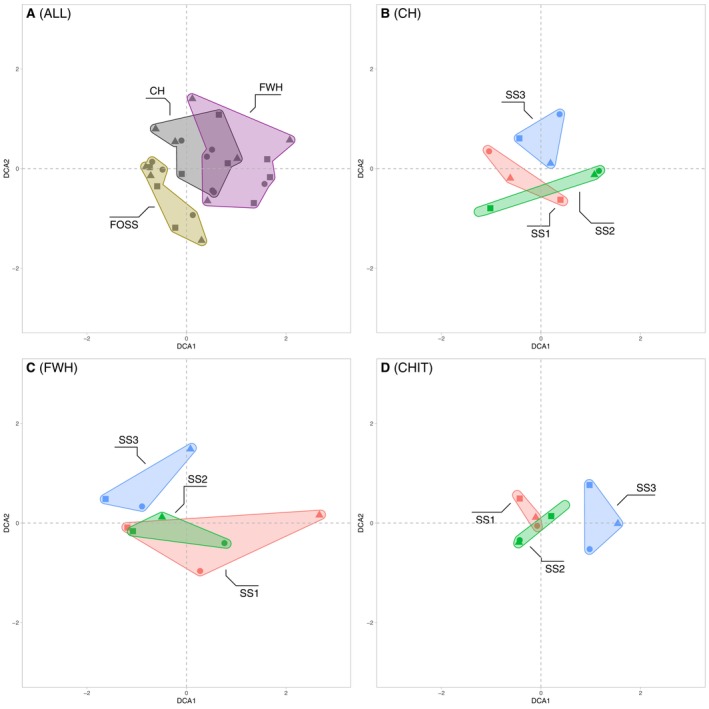
Ordinations of Chironomidae morphotypes per sediment core and method. The DCA analyses show the clustering of samples according to the methodological approaches (ALL) and specific to a particular method (CH = Chironomidae‐specific primers, FWH = invertebrate‐universal primers and CHIT = chitinous remains counts). All abundance data were presence–absence transformed before ordination. Symbols represent the core layer samples (circle = bottom, triangle = middle and square = top). CH‐ and FWH‐based identifications were translated to the corresponding chitinous remain morphotype for comparing the results.

The comparison between the morphotype‐assigned metabarcoding Chironomidae taxa and the detections based on chitinous remains showed some overlap but, overall, a method‐specific morphotype composition (Figure 6A). The DCA analysis revealed that the two molecular approaches with primer combinations CH and FWH have relatively similar chironomid morphotype compositions for the examined sediment samples (Figure 6A). Furthermore, closer clustering and therefore higher similarity of species assemblages of the chironomid‐specific primer set CH, and CHIT was apparent compared to the more universal primer set FWH and CHIT. Therefore, results indicated more shared morphotypes between CH and CHIT when species identifications were translated to chitinous remains morphotypes (Figure 6A). DCAs comparing morphotype assemblages based on either CH, FWH or CHIT for the samples originating from the different cores consistently showed a higher degree of morphotype composition similarity and more overlap in the DCA biplot sample scores between shallow‐depth sediment cores SS1 and SS2 samples but more pronounced differences with the deep‐lake core SS3 (Figure 6B–D).

**FIGURE 7:**
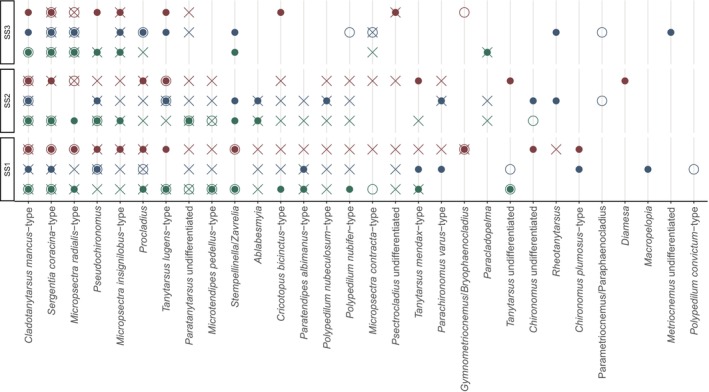
Overview of Chironomidae morphotypes recorded with sedDNA metabarcoding and chitinous remains identification. The filled circles represent CH‐detections, the empty circles FWH‐detections and the crosses indicate the presence of the morphotype in the chitinous remains dataset (CHIT). Bottom sediment core layers are shown in green, middle layers in blue and top layers in red. Read abundances and remain counts were presence–absence transformed. CH‐ and FWH‐based identifications were translated to the corresponding chitinous remain morphotype for comparing the results.

Among the shared morphotype‐assigned metabarcoding‐derived taxa and the chitinous remains Chironomidae morphotypes, *Sergentia coracina*‐type and *Micropsectra radialis*‐type were consistently recorded with all three methods (CH, FWH and CHIT) in a number of samples and were present in all sediment cores (Figure 7). However, some morphotypes show less consistent patterns. For example, *Tanytarsus lugens*‐type was detected both based on CHIT and with the sedDNA‐based approaches CH and FWH, but not consistently in the same samples and cores and was, e.g., not detected with CHIT or FHW in SS3 (Figure 7). Other taxa, e.g., *Paratanytarsus* and *Microtendipes pedellus*‐type, were consistently found with CHIT in most samples but only occasionally with CH or FHW. *Cladotanytarsus mancus* type was found in two cores with CHIT and consistently in all three cores with CH and for some samples also with FHW. Records in SS3 with CH showed the presence of the species in all samples even though chitinous remains were not found (Figure 7). Other individual identifications (e.g., *Chironomus plumosus*‐type, *Maropelopia* and *Polypedilum convictum*‐type) were detected based either on CH or FHW but not the other approaches (Figure 7). In contrast to the chironomid morphotype‐specific method comparison, the metabarcoding datasets containing all invertebrate morphotype assignments showed considerably less core specificity (Figure S17) when compared to the CHIT dataset (Figure 2).

## 4 Discussion

Lake sediments contain remains of a wide range of aquatic organism groups including different types of algae (e.g., diatoms, cyanobacteria or green algae) but also a wide range of arthropods such as crustaceans (e.g., Ostracoda, Cladocera; Smol, Birks, and Last 2001) and aquatic insects (e.g., Trichoptera, Ephemeroptera, Diptera; Courtney‐Mustaphi et al. 2024). Chironomidae are often among the most abundant invertebrate remains preserved in lake sediments (e.g., Ursenbacher, Stötter, and Heiri 2020). These remains can provide information on past Chironomidae diversity changes (e.g., Engels et al. 2020) and indirectly indicate past alterations in environmental conditions (Bolland et al. 2021; Brodersen and Quinlan 2006; Verbruggen et al. 2011). Recently deposited chironomid remains in lake surface sediments have been analysed to assess the distribution of chironomid assemblages relative to environmental variables such as summer temperature (e.g., Heiri et al. 2011), nutrient concentrations (e.g., Lotter et al. 1998) or deep‐water oxygen availability (e.g., Luoto and Salonen 2010). Methodologically, the identification of Chironomidae remains relies primarily on the morphological assignment of chitinous remains to morpho‐groups. Aside from this relatively well‐established approach, new method developments based on sedimentary DNA are still unexplored for determining past and present chironomid assemblage compositions, despite the potential already shown by previous studies for other organism groups (Domaizon et al. 2017; Garcés‐Pastor et al. 2022; Li et al. 2023).

To bridge this gap, we explored the suitability of sedDNA metabarcoding based on an invertebrate‐universal (FWH) and a chironomid‐specific (CH) primer combination and compared the obtained data with traditional morphotype identifications. We focused on sub‐recent sediments from different depths of short sediment cores obtained from a Swiss lowland lake in which syn‐ and post‐diagenetic alteration of DNA should not (surface sample) or not entirely (middle and low sample) have taken place. The results allowed us a first‐order assessment of whether FWH and CH can isolate chironomid sedDNA from the samples, whether the results are comparable to identifications based on chitinous chironomid remains, and whether metabarcoding and organism remains‐based approaches can detect similar patterns of chironomid species compositions.

Overall, results showed that Chironomidae can be detected with both approaches, FWH and CH. However, the chironomid‐specific primer set detected considerably more chironomid taxa (33 species) than the FWH primer set (19 species). In contrast, FWH detected DNA from a broader taxonomic range, e.g., including Mollusca or Cnidaria assigned ESVs (see Section 3.2). This indicates substantial primer bias and the need to carefully consider metabarcoding method combinations when designing studies targeting specific taxa in lake sediment samples. Nonetheless, both approaches detected DNA of fossil Chironomidae in all the examined samples, including sediment samples obtained from as low as 35 cm below the sediment–water interface.

The comparison between the fossil count and sedDNA datasets using DCA revealed comparable overall similarity patterns between the samples, particularly between sediment cores obtained at different depths within the lake basin. The results show a distinct faunistic composition of the deep lake sediment core SS3 compared to the more similar shallow depth SS1 and SS2 cores based on all three approaches. This indicates that the overall similarity patterns of the datasets obtained with the different methodological approaches was comparable. Nonetheless, considerable differences are apparent between the taxonomic assignations based on the three approaches. Compared to the two sedDNA metabarcoding approaches (see Figure 5), we detected considerably more Chironomidae taxa with the traditional fossil remain identifications, even when taxonomic records were translated to the same taxonomic resolution (i.e., taxa identified using FWH and CH were adjusted to the respective morphotypes detectable based on chitinous remains). For example, *Corynoneura edwardsi*‐type was detected as a chitinous remain, but no equivalent species was found based on CH or FWH (see Tables S11 and S12). Similarly, some taxa were frequently recorded based on the analysis of the remains in the sediment samples (e.g., *Microtendipes*), but only rarely and in individual samples with CH or FWH.

These differences can have several underlying causes and processes with varying influence. One of the major well‐known influencing factors in metabarcoding studies is the quality and completeness of reference sequence databases (Blackman et al. 2019, 2022; Keck, Couton, and Altermatt 2023). Recent Chironomidae CO1‐Barcoding studies report rather underrepresented species coverage in commonly used sequence databases such as BOLD or NCBI GenBank (Gadawski et al. 2022; Lencioni, Rodriguez‐Prieto, and Allegrucci 2021; Lin, Stur, and Ekrem 2017). Combined with our relatively stringent sequence identification and assignment strategy, this excludes all ESVs without a database match close to the species level. Especially when lacking reference sequences for a species or sequences with high intraspecific variability, this can result in a high proportion of unidentified ESVs. Consequently, such ESVs were not assigned to a valid species name; hence, they were not translated into an equivalent morphotype or included in our comparison with detected chitinous remains. However, future efforts to add more chironomid species to reference sequence databases would certainly improve the detection rate and lead to more complete Chironomidae community reconstructions. Efforts focused on COI‐Barcoding of species present in a particular target region or ecosystem type (e.g., in our case Lake Sempach or similar Swiss lowland lakes in general) before future studies could significantly increase the number of chironomid species detections and improve overall comparability with chitinous remains‐derived datasets.

Furthermore, the quality of the taxonomic sequence annotations in the reference databases plays a vital role (Keck, Couton, and Altermatt 2023). In the present study, we decided to remove species information with typical signs of uncertainty, such as nomenclature containing *sp*., *agg*. or *aff*., and set the respective taxonomic annotations back to the lower taxonomic level, gaining confidence but losing species‐level information. Hence, assigning such ESVs to a chitinous remain morphotype can be difficult and influence final information recovery because remains‐derived morphotypes usually comprise several recent species. Another aspect influencing species detections is the minimum number of reads threshold chosen during the read‐to ESV mapping process. We stringently filtered and quality‐checked the raw sequencing reads to ensure true biological information and avoid technical bias. Given the general aim of this study and expecting rare species with low sedDNA abundance (see, e.g., Capo et al. 2021; Capo, Barouillet, and Smol 2023) in the case of Chironomidae, we allowed for low read‐abundance detections. However, this might not be ideal in another scenario, e.g., if relative quantification is important or if there is a high risk of sample contamination.

Finally, for many Chironomidae species, the larval morphology remains undescribed. For example, several species‐level morphotypes have been described for the genus *Micropsectra*. However, the morphotype assignment of *Micropsectra notescens* (Walker, 1856), which we identified with CH, still needs to be clarified. The description of larval characteristics of further species, particularly their head capsule structures that preserve well as remains in the sediments (e.g., the mentum, ventromental plates, antennal pedestals, mandibles or relevant pore positions), would therefore also allow a more detailed and reliable comparison of chironomid morphotypes based on chitinous remains with metabarcoding‐derived identifications.

Sample standardisation is another essential aspect that should be considered carefully concerning future method comparison studies. For sedDNA samples, a precise amount of sediment volume or mass is usually processed. This sediment may, in principle, contain DNA fragments from many individuals. In contrast, the processing of chitinous remains usually relies on reaching a minimum count of individuals in samples with limited numbers of remains, often 45–50 Chironomidae head capsules (Heiri and Lotter 2001; Larocque 2001; Quinlan and Smol 2001b). This number has been proposed to obtain reliable estimates of the abundance of the most dominant chironomid morphotypes and for ordinations or inferences based on regression‐type models. However, a minimum count of 50 may not adequately represent rarer taxa, which could be detected with sedDNA metabarcoding approaches. This effect could, e.g., explain why several species were detected as single occurrences only by CH or FWH, whereas these presumably rare species were not detected based on counts of organism remains. Therefore, future chitinous remains count data comparisons with metabarcoding‐based species detections may consider raising the minimum count to higher levels (e.g., 100 or 150 chironomid head capsules) to better represent rarer chironomid species in these analyses. Overall, we aimed to provide first evidence of the potential applicability of sedDNA metabarcoding to detect chironomid assemblages and did not focus on in‐depth method assessments with both approaches. However, further assessing intra‐method variability, e.g., sample replication and its influence on the taxonomic composition, with specific experimental designs would be an essential next step prior to further method comparisons and implementation of sedDNA metabarcoding targeting chironomidae (see also Capo et al. 2021; Capo, Barouillet, and Smol 2023). Another aspect of method development would be field‐negative control standardisation. We see our approach with an open tube that would capture contaminating recent DNA while cutting a sediment core layer as a good starting point. With this strategy, one could identify potentially contaminating DNA from sources such as surrounding air or the equipment used. The only taxon match of a field‐control derived ESV with the reference database was *Hydra virdissima*, a common freshwater Hydrozoan. In addition, a BLAST search revealed the presence of a few *Bos taurus* and *Hyaloperonospora* DNA reads, both very abundant and likely to be transported by wind around a freshwater lake environment. None of these field‐control reads matched a taxon relevant to our results. However, it shows the possibility of the presence of contaminating DNA in such an environment, and finding a fully optimised field‐negative control process should be further developed and improved, especially in studies that address very low‐abundance targets. Irrespective of these methodological challenges, which can at least be partially resolved by improving reference database quality, taxonomic coverage in reference databases and their curation, as well as addressing sample standardisation and further method assessment, our results showed the overall applicability of sedDNA metabarcoding in lake sediment studies focusing on Chironomidae.

In this study, we analysed recent and subrecent samples. However, a further obvious application of a sedDNA metabarcoding approach for identifying Chironomidae samples would be exploring truly ancient sedDNA from longer sediment sequences encompassing extended sections of the sedimentation history of lakes. Similar studies targeting plant or mammal DNA have recently been conducted (e.g., Garcés‐Pastor et al. 2022; Gauthier et al. 2022; Giguet‐Covex et al. 2014) and have shown that sedDNA analyses of lake sediment samples can provide information on past vegetation change, species assemblage compositions and past presence of livestock and other grazers in the vicinity of lakes.

Despite our focus on more recent sediment samples, our results show that Chironomidae DNA is detectable in lake sediments well below the water–sediment interface. More importantly, they confirm that metabarcoding‐based analyses of chironomid species assemblages can detect a comparable similarity structure between our lake sediment samples as the conventional approach, even considering the uncertainties affecting our results, such as the underrepresentation of Chironomidae sequences in reference databases.

Although sedDNA has considerable potential for reconstructing past changes in Chironomidae composition as a stand‐alone approach, our results also indicate that sedDNA‐based and chitinous remain‐based analyses can provide complementary information on the past composition of Chironomidae communities in lakes. Our first findings indicate that the approaches can detect more Chironomidae taxa together than individually, suggesting that some taxa are more likely to be detected and identified by one approach than the other. Morphotype analysis with chitinous remains tends to provide robust estimates of the abundances of chironomid larvae, which can, e.g. also provide information on past changes in climatic conditions, oxygen conditions or trophic states of lakes (e.g., Heiri et al. 2014; Verbruggen et al. 2011). In contrast, reconstructing past abundances with sedDNA metabarcoding is more challenging due to uncertainties of the comparability of sequence read abundance and the actual number of individuals (e.g., Elbrecht and Leese 2015). However, a major advantage of sedDNA metabarcoding is that it allows for species‐level taxonomic information compared to morphotype analysis, which usually identifies chitinous remains to the species group or genus level rather than the species level (Brooks, Langdon, and Heiri 2007). Therefore, we expect considerable potential for using the combined and parallel methods in future studies focusing on surface sediment samples and downcore sediment records. The morphological identification of remains can provide robust estimates of the abundances of the dominating chironomid groups at a particular coring location, and the metabarcoding approach could complement this information with detailed data on the species included in the different morphotypes. A combined approach may allow for an improved and more intuitive comparison of lake sediment‐based assessments of chironomid species assemblages. In combination with ecological information based on neo‐ecological surveys of chironomid larvae in lakes, this would benefit bioindicator performance to assess environmental conditions in lakes based on indicator systems developed for macroinvertebrate samples, such as the Benthic Quality Index (Jyväsjärvi, Aroviita, and Hämäläinen 2014) or the Saprobic index (Rolauffs et al. 2004).

To conclude, our results show that the analysis of morphological remains and metabarcoding‐based assessments can successfully produce complementary information on the assemblage composition of Chironomidae species based on lake sediment samples. The fine‐scale taxonomic information, however, differs between the two approaches and further investigations on specific factors influencing sedDNA metabarcoding taxa recovery, e.g. sample standardisation, amount of sediment, reference database quality and species coverage, are needed to improve comparability and further develop the methods. We suggest that future method developments should include (1) enhancing Chironomidae species reference sequence databases with a focus on regions and lakes of interest; (2) comparing metabarcoding‐based assessments and identifications based on morphological remains with a higher number of surface sediment samples representing different depositional and environmental conditions, to compare the methods over a more extensive environmental range and (3) should apply the combined methods to downcore sediment records representing larger and older time intervals, to assess whether they can provide complementary information on past changes in chironomid assemblages and in‐lake environments.

## Author Contributions

L.A.B. designed the study, planned fieldwork, conducted the molecular lab work, bioinformatically processed the sequences, analysed the data and wrote the original draft of the manuscript. P.L. contributed to field and lab work, isolated the chitinous remains, conducted the morphological identification and contributed to the writing process. C.C.‐M. contributed to the fieldwork and was involved in writing the manuscript. O.H. secured the financing of the study, contributed to the study design and data analysis and did the main editing and reviewing of the manuscript.

## Conflicts of Interest

The authors declare no conflicts of interest.

## Supporting information


Data S1.

Data S2.

## Data Availability

Raw sequences of the metabarcoding dataset produced in the present study are available in the NCBI SRA database under the BioProject accession PRJNA1062313.
