# Supplementary material for: Sediment Core DNA‐Metabarcoding and Chitinous Remain Identification: Integrating Complementary Methods to Characterise Chironomidae Biodiversity in Lake Sediment Archives
Source: Mol Ecol Resour. 2024 Oct 21;25(1):e14035. doi: 10.1111/1755-0998.14035 (PMC11646301; doi:10.1111/1755-0998.14035)
Supplement: Supplementary file 1 — Data S1. [file MEN-25-e14035-s002.docx]

**Supplemental Information for:**

**Sediment core DNA-Metabarcoding and chitinous remain identification: Integrating complementary methods to characterise Chironomidae biodiversity in lake sediment archives**

Lucas André Blattner, Pierre Lapellegerie, Colin Courtney-Mustaphi and Oliver Heiri

| **Table of Contents:** |  |
| --- | --- |
| **Figure S1** | Page 1 |
| **Figure S3** | Page 2 |
| **Figure S10** | Page 3 |
| **Figure S13** | Page 4 |
| **Figure S15** | Page 5 |
| **Figure S17** | Page 6 |

**Figure S1: DNA yield per extraction method and sediment weight.**

The DNA concentration in 100 µl TE (low EDTA) buffer is shown per extraction kit (A; n = 18 per extraction kit) and amount of extracted sediment (B; n = 27 per sediment mass). Extraction methods are abbreviated with FastDNA for the FastDNA^TM^ Spin Kit for Soil, PowerSoil for the DNeasy PowerSoil Pro kit, and PowerSoilPhos followed the same procedure as DNeasy PowerSoil with the addition of 1 M phosphate buffer.

**Figure S3: Genetic pairwise similarity between chironomid CO1 sequences**

The heatmap shows the pairwise distances between the downloaded 139 bp long chironomid CO1 insert sequences. Sequence divergence ≤ 0.08 (≤ 8%) is coloured in orange.

**Figure S10: Chironomidae and other invertebrate chitinous remains morphotype detections.**

Columns represent the different cores (SS1, SS2 and SS3. The circles are coloured according to the core layers (bottom = green, middle = blue and top = red), and circle sizes represent the relative number of isolated chitinous remains. The exact remain counts are documented in supplementary Table S10.

**Figure S13: Method-specific comparison of ESV diversity**

**Figure S15: Chironomidae and other invertebrate sedDNA detections**

Columns represent the different cores (SS1, SS2 and SS3. The symbols show the different primer sets and are coloured according to the core layers (rhombus shapes = FWH, circles = CH; bottom = green, middle = blue and top = red). Symbol sizes represent the number of reads assigned to the respective taxa. The exact read abundances are documented in supplementary Tables S6, S7 and S11.

**Figure S17: DCA ordinations of the metabarcoding datasets containing all invertebrate taxa**

The detrended correspondence analysis for the chironomid-specific CH (A) and the invertebrate-universal FWH (B) primer sets shows the clustering of samples specific to the two littoral cores SS1 and SS2 and the deep lake core SS3.
